# Reporting Experiments in Homeopathic Basic Research—Description of the Checklist Development

**DOI:** 10.1093/ecam/nep170

**Published:** 2011-03-10

**Authors:** B. Stock-Schröer, H. Albrecht, L. Betti, G. Dobos, C. Endler, K. Linde, R. Lüdtke, F. Musial, R. van Wijk, C. Witt, S. Baumgartner

**Affiliations:** ^1^Karl and Veronica Carstens-Foundation, D-Essen, Germany; ^2^Department of Agro-Environmental Science and Technology, Faculty of Agriculture, Bologna University, I-Bologna, Italy; ^3^Chair of Complementary and Integrative Medicine, University of Duisburg-Essen, D-Essen, Germany; ^4^Interuniversity College for Health and Development, A-Graz, Austria; ^5^Institute of General Practice, Technical University, D-Munich, Germany; ^6^International Institute of Biophysics, D-Neuss, Germany; ^7^Institute for Social Medicine, Epidemiology and Health Economics, Charité University Medical Center, D-Berlin, Germany; ^8^Institute of Complementary Medicine KIKOM, University of Bern, CH-Bern, Switzerland

## Abstract

The objective of this study was to develop a criteria catalogue serving as a guideline for authors to improve quality of reporting experiments in basic research in homeopathy. A Delphi Process was initiated including three rounds of adjusting and phrasing plus two consensus conferences. European researchers who published experimental work within the last 5 years were involved. A checklist for authors provide a catalogue with 23 criteria. The “Introduction” should focus on underlying hypotheses, the homeopathic principle investigated and state if experiments are exploratory or confirmatory. “Materials and methods” should comprise information on object of investigation, experimental setup, parameters, intervention and statistical methods. A more detailed description on the homeopathic substances, for example, manufacture, dilution method, starting point of dilution is required. A further result of the Delphi process is to raise scientists' awareness of reporting blinding, allocation, replication, quality control and system performance controls. The part “Results” should provide the exact number of treated units per setting which were included in each analysis and state missing samples and drop outs. Results presented in tables and figures are as important as appropriate measures of effect size, uncertainty and probability. “Discussion” in a report should depict more than a general interpretation of results in the context of current evidence but also limitations and an appraisal of aptitude for the chosen experimental model. Authors of homeopathic basic research publications are encouraged to apply our checklist when preparing their manuscripts. Feedback is encouraged on applicability, strength and limitations of the list to enable future revisions.

## 1. Introduction

Homeopathy is a widely used and highly controversial complementary therapy. The central aspect of the dispute is the use of highly diluted, “potentized" remedies which, according to conventional scientific thinking, make any biological activity highly unlikely. But also the validity of the fundamental tenet of homeopathy—the Similia Principle (like cures like)—is not generally accepted by the scientific community. Therefore, any publication of a research study in homeopathy, particularly if reporting positive results, is subjected to special scrutiny. Clinical research is of utmost importance to investigate efficacy and effectiveness. Basic research is needed to investigate possible mechanisms of action. However, as clinical research so far has failed to prove or disprove specific effects of homeopathy in general and of high potencies in particular, laboratory research is also needed to investigate whether homeopathic preparations have biological activity.

Published experiments can be assigned to four main areas of basic research on homeopathy: animal research, plant bioassays, *in vitro* models and physicochemical research [1]. The HomBrex Database lists more than 1100 experiments published in about 900 publications in different fields of basic research [2]. To appreciate and understand the methods and findings of such studies, a high degree of transparency is required from authors. Reviews have shown that many experiments differ in design and quality [3]. Over the years, several efforts have been made to enhance the quality of basic research in homeopathy. Schulte proposed a set of standards for ultra high dilution research [4], Linde et al. worked out checklists for preparing and reviewing publications [5] and also suggestions on methodological standards [6] have been published. Moreover scoring systems for assessing quality were generated: the first for the use in experimental toxicology [7] the second for physicochemical research into homeopathic potentization [8]. The latter score was modified and used in a systematic review on *in vitro* experiments [3].

It seemed reasonable to try to bring together all aspects of former evaluation scores and to develop with a team of experienced researchers a basic score applicable (possibly in a slightly adjusted format) in all fields of basic research on homeopathy. During the discussion process it was decided to shift the focus from the development of a scoring system to the development of a reporting checklist for authors, peer reviewers and editors (and possibly readers and authors of systematic reviews) similar to publication guidelines in clinical research (CONSORT, REDHOT). This article describes and discusses the development process of the checklist (REHBaR—Reporting Experiments in Homeopathic Basic Research). A second publication will focus on the last step of the Delphi Process (round V), where the discussed criteria and their detailed explanations as a result of the whole process are given, supported and further evidenced from other publications.

## 2. Methods

### 2.1. The Delphi Process: A Consensus Method

From the existing consensus methods we chose a Delphi approach. Delphi may be characterized as a method for structuring a group communication process so that the process is effective in allowing a group of individuals, as a whole, to deal with a complex problem. In order to obtain a useful result for their objective there is a need to structure a group communication process [9]. This Delphi technique is applicable to a variety of questions ending up with different outcomes. In our case, the goal was to create an agreed checklist with criteria important for improving the quality of reports in basic research. A search for papers in the databases PubMed and CAMbase with the following keywords was conducted until July 2009: guideline, reporting, publication, catalogue, basic, clinical, fundamental, research, author, quality, CONSORT.

### 2.2. Preparatory Work

Before starting the Delphi process a preliminary collection of 58 potentially relevant items was compiled as preparatory work. The collection comprised all items included in the two scores developed for systematic reviews in basic research [7, 8]. Additional items were included on the basis of informal discussion with experts and the corresponding author.

### 2.3. The Delphi Process (Round I–V)

**Figure 1:**
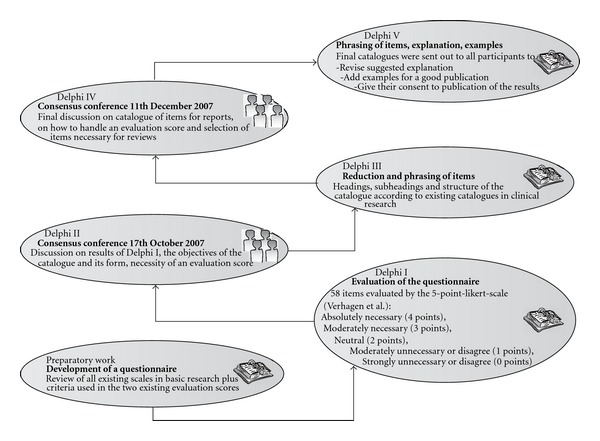
Flow chart of the Delphi process realized from October 2007 to March 2009 among a group of European research on homeopathy for developing the checklist REHBaR.

In the Delphi process there were a total of five rounds, including two consensus conferences (see Figure 1). In Delphi I, the list of 58 items primed in the preparatory phase by the initiators was sent to all participants. All experts were asked to rate the importance of each item on a 5-point Likert scale, and to add further items. Delphi II—the first conference—offered the opportunity to clarify misunderstandings and misconceptions and discuss the objective of the catalogue in general. In Delphi III a modified catalogue was prepared taking into account the revised objective (see “Results"), which was again discussed in the conference Delphi IV. In Delphi V, the reporting document was prepared and iteratively revised in an internal review process.

### 2.4. Team of Experts

Members of the panel were S. Baumgartner, L. Betti, C. Endler and R. van Wijk as leading European researchers in homeopathic experiments with plants, animals, evolutionary biology as well as biochemical and biophysical research. K. Linde and C. Witt were involved in Delphi for two reasons: first they are well known experts in the field of clinical research concerned with quality questions, and secondly both are experienced in basic research and developed a score for quality assessment in basic research [7, 8]. R. Lüdtke was responsible for the statistical aspects, H. Albrecht, G. Dobos and F. Musial for general support in questions of basic research.

## 3. Results

### 3.1. Delphi Round I: Rating and Selection of Items

In the first round, all members of the team rated the relevance of the 58 items included in the preliminary list. Out of 58 items, 18 were rated as “absolutely necessary" (mean: 3.5–4) and 31 items as “moderately necessary" (mean: 2.5–3). No item was rated “moderately unnecessary" or “strongly unnecessary", but nine items received neutral rating (mean: 2). Only one item was added and discussed in the conference.

### 3.2. Delphi Round II: The First Consensus Conference

In the first conference we came to the conclusion that our initial intention—creating a new evaluation score—should be postponed. A criteria catalogue for improving the quality of reporting experiments was considered a priority. The main reason for this was that accurate reporting is a precondition for a valid quality assessment. Furthermore, the importance of quality scoring, in clinical research, is currently under discussion. For example, the current version of the Handbook of the Cochrane Collaboration, a worldwide network for performing systematic reviews on clinical research, explicitly discourages the use of quality scores [10]: it is argued that while this method of scoring is simple, it is not supported by empirical evidence. The calculation of summary scores involves assigning “weights" to different items in the scale, and it is difficult to justify the weights assigned. Furthermore, scales have been shown to be unreliable assessments of validity [11]. Therefore it is regarded as preferable to use simple approaches for assessing validity that can be fully reported (i.e., how each trial was rated on each criterion).

### 3.3. Delphi Round III: Phrasing of the Checklist

As a result, a checklist for adequate reporting was then compiled and worded in this round following existing examples in clinical research, mainly CONSORT [12] and STROBE [13].

### 3.4. Delphi Round IV: The Second Consensus Conference

Another conference was held in order to discuss the reassessed first checklist looking at the purposes of exploratory and confirmatory experiments. The final list, unanimously agreed upon, is presented in Table 1: “Items to be included when Reporting Experiments in Homeopathic Basic Research". It was then decided to refrain from establishing an evaluation score. As an additional step, the group selected those items from the first list that were considered to be the minimum essential information needed for reviewing publications in this field. This new catalogue includes all parts readers should know to clearly follow the experiments and to fully understand the results. The catalogue will be published after an internal reviewing process and test of practicability.

### 3.5. Delphi Round V: Rewording of the Checklist Supplemented with Explanations

In the last round detailed explanations for each item based on the first catalogue were written, reassessed by all participants and supplemented with examples (available from the corresponding author). Often it is not possible to give all information due to space limitations of the journal. Therefore we recommend authors to refer to a website address to make further information available. If certain methods or the procedure of positive and negative controls are already published, it may be sufficient to refer to that publication. As publications often report on several experiments performed with regard to one research question, the report should distinguish between the single experiments and make them obvious to the reader. Furthermore, it is important to make clear if the experiment was designed for explorative or confirmatory purpose and whether it followed a specific hypothesis or not.

## 4. Discussion

Accurate reporting is a prerequisite for critical interpretation of any research study and its findings. Without accurate reporting it is not possible to assess the merits of a study. Any positive findings of basic research experiments on high dilutions or potencies are likely to stir controversy in the academic community. If details in the publication (or an additional accessible report) are insufficient to allow repetition of experiments performed or to assess potential threats to validity, the value of a potentially important experiment is strongly compromised. In an 18-month Delphi process we have tried to come up with a checklist aimed at helping authors to prepare high quality manuscripts on their homeopathic basic research experiments, and at helping peer reviewers, editors and readers to check whether reporting accurately reflects their experiments.

Shortcomings in reporting experiments are not at all unique to homeopathy but a general phenomenon. The limited space in journals, specific style and editorial pressure often force authors to shorten their paper to an extent where a detailed description becomes impossible. However, even with limited space, a good manuscript can provide a lot of relevant details, and the internet makes it possible to make more detailed reports easily available to interested researchers. In clinical and epidemiological research several checklists to improve reporting quality have been developed within the last number of years. The first attempt to improve quality of reporting randomized clinical trials was the CONSORT statement in 1996 [14]. This document has been revised in 2001 [12] and 2005 [15], and recently further documents have become available for improving the reporting of abstracts [16], pragmatic trials [17], non-pharmacological trials [18] (see also http://www.consort-statement.org/). The necessity of CONSORT was recently encouraged by the poor standards common in reporting RCTS in Tai Chi interventions [19] as well as the poor standard of reporting in Chinese journals [20].

QUORUM provides a guideline for reporting meta-analyses [21, 22]. An evolution of this guideline was developed by an international group: Preferred Reporting Items for Systematic reviews and Meta-Analyses (PRISMA) [23]. For observational studies, including cohort, case-control and cross-sectional studies STROBE emerged [13], recently extended with STREGA (STRengthening the Reporting of Genetic Association studies) [24]. Comparable lists are available for trials in acupuncture: STRICTA [25] is already discussed [26] and assessed [27]. For complete and accurate reporting of studies of diagnostic accuracy (STARD) an item list was compiled [28]. Standards for Quality Improvement Reporting Excellence (SQUIRE) were published to enhance reporting on quality improvement studies in health care [29]. RedHot [30], an unofficial extension for CONSORT, was established to assist reporting on homeopathic treatments in clinical trials. A further CONSORT statement was compiled [31] concerning randomized, controlled trials of herbal interventions, evaluated in a systematic review of instruments developed to critically assess the quality of trials on the efficacy of natural health products [32]. A complete collection of available reporting guidelines can be found at EQUATOR (Enhancing the QUAlity and Transparency Of health Research), a new international initiative based on a network concept [33].

Basic research is different from clinical and epidemiological research, and reporting guidelines comparable to those described above for clinical research do not exist in this area outside of homeopathy. In biological sciences there are some groups working on standards of data presentation. These initiatives were derived from research groups dealing with microarray experiments and provide several checklists on how to report and deal with large numbers of data [34]. An overview of these activities can be found at the homepage of MIBBI (Minimum Information for Biological and Biomedical Investigations) http://www.mibbi.org or the MGED (*Microarray Gene Expression Data*) society.

Our checklist is compiled for experiments on homeopathy and how to report each step of experiment which is important to understand and appreciate the results.

A first proposal for a reporting guideline in homeopathy was introduced in 1991 [35]. It focused on experiments dealing with ultra low dose effects (serial dilutions and potencies).

The group refrained from establishing a score for assessing quality, as had been common in the past, in clinical research [36]. In the current state it was thought that it is not feasible to propose clear-cut criteria for assessing what constitutes good and bad basic research beyond generally accepted standards. Furthermore, a lot of basic research experiments are explorative and often procedures cannot follow a predictable outline. Research questions tend to be more complex than the methodologically straightforward question of efficacy. Unexpected effects are common, either due to the potentization process, the level of potentization or the substance itself. In exploratory experiments it is often not obvious to which part of the intervention the effect refers to and to what extent. A precise description of the manufacturing and the Pharmacopoeias of the test and control substances are therefore indispensable. If the expected effect is caused by the succussion process only, unpotentized solvent is the adequate control. If the effect is expected to be caused by a potentized substance, a potentized solvent or another potentized substance (at the same potency level) is adequate. When investigation was performed on the Similia Principle, a variety of substances, which represent different degrees of similarity with the diseased state, can be chosen as control. It is important to explain why which control was selected relating to the underlying questions of research, for example, the Similia Principle or Isopathy. In this context the role of individualization in experiments performed about the Similia Principle or the definition of Isopathy should be taken into account.

Whether quality assessment scores make sense in the future remains to be discussed. In clinical research there is a clear trend to assess single components of quality and investigate their impact on outcomes instead of using questionable summary scores. “One commonly-used scale was developed by Jadad and colleagues for randomized trials in pain research [37]. The use of this scale is explicitly discouraged. As well as suffering from the generic problems of scales, it has a strong emphasis on reporting rather than conduct, and does not cover one of the most important potential biases in randomized trials, namely allocation concealment." (Cochrane Handbook, Chapter 8.3.3) [10].

REHBaR was developed with a standard consensus method among an international team of researchers with experience in basic and clinical research in homeopathy, experimental physiology, general research methodology and statistics. All members commented on intermediate and the final version of the checklist. Obviously, the team was a relatively small sample of individuals, and other researchers might have introduced other items. The current list has to be considered as a first try and it is hoped that it proves useful to enhance the quality of reporting basic research experiments in homeopathy. We encourage the use of the list, critical feedback and hope to be able to provide an improved version in a few years.

## Figures and Tables

**Table 1:** Items to be included when reporting experiments in REHBaR.

Item	No.	Descriptor
Title	1	Title indicates the experimental model and intervention
Abstract	2	Abstract provides an informative and balanced summary of what was done and found

Introduction		
Background	3	Scientific background, presentation of experimental model(s). explanation of rationale, including homeopathic principles (e.g., similia principle, potentization, proving) and type of homeopathy (isopathy, classical versus complex homeopathy)
Objectives/Hypotheses	4	Objectives and hypotheses with outcome measures. For confirmatory experiments: specific hypotheses and clearly defined primary outcome measure. For exploratory experiments: hypotheses inducing the investigations

Materials and methods		
Materials	5	Detailed description of all used materials (e.g., biological system, devices, substances, instruments)
Materials (homeopathy specific)	6	Manufacturer, pharmacopoeia (or process) of medications, potency and steps of dilution, dilution method, substance starting point of dilution (e.g., mother tincture. D1, nosode)
Homeopathic controls	7	Precise details on the preparation of the control substance
System performance controls	8	Report on negative and positive controls
Quality control	9	Procedures and efforts used to enhance the quality and reliability of the experimental procedure
Object of investigation	10	Selection criteria for the particular system used: *in vivo*, *in vitro*, biological, physical, biochemical
Experimental setup	11	Detailed description of experimental conditions and procedure
Replication	21	If experiment has internal replications, detailed description is given of which materials were reused and which have been changed
Parameters	13	All measured parameters described in detail
Intervention	14	Precise details of the interventions intended for each group and how and when they were actually administered
Allocation	15	Method used to generate the group allocation including details (e.g., randomization, blocking, stratification)
Blinding	16	Description if any procedures or interventions were concealed (if yes, details given)
Statistical methods	17	Statistical tests and procedure of calculation are described: Methods for additional analyses like adjusted analyses

Results		
Numbers analysed	18	Number of experiments with exact number of treated units per setting which were included in each analysis and reporting missing samples, drop outs
Data (descriptive)	19	Results are given in tables or figures showing mean or median together with variability (e.g., SD and/or range) for absolute data (and differences)
Data (inferential)	20	Gives appropriate measures of effect size uncertainty and probability
Discussion		
Interpretation	21	Interpretation of the results, taking into account study hypotheses, sources of potential bias or imprecision
Evidence	22	General interpretation of results in the context of current evidence. Discuss the generalizability/external validity of the study results
Experimental model	23	Explanation why this model, these parameters were chosen and its adequacy for answering the questions including homeopathic aspects
